# Selective laser trabeculoplasty for early glaucoma: analysis of success predictors and adjusted laser outcomes based on the untreated fellow eye

**DOI:** 10.1186/s12886-016-0385-z

**Published:** 2016-11-23

**Authors:** Mikael Chun, Carolina P. B. Gracitelli, Flavio S. Lopes, Luis G. Biteli, Michele Ushida, Tiago S. Prata

**Affiliations:** 1 Department of Ophthalmology, Federal University of São Paulo, Rua Botucatu, 821. Vila Clementino, São Paulo, São Paulo CEP: 04023-062 Brazil; 2 Glaucoma Unit, Hospital Medicina dos Olhos, Osasco, São Paulo Brazil

**Keywords:** Selective laser trabeculoplasty, Intraocular pressure, Fellow eye, Open-angle glaucoma, Treatment

## Abstract

**Background:**

To identify success predictors and to study the role of the fellow untreated eye as a co-variable for adjustment of intraocular pressure (IOP) outcomes following selective laser trabeculoplasty (SLT) in early open-angle glaucoma (OAG) patients.

**Methods:**

A case series was carried out. Patients with uncontrolled early OAG or ocular hypertension (inadequate IOP control requiring additional treatment) underwent SLT (one single laser session) performed by the same surgeon in a standardized fashion. The same preoperative medical regimen was maintained during follow-up for all patients. Post-treatment assessments were scheduled at week 1 and months 1, 2, and 3. In order to account for possible influence of IOP fluctuation on laser outcomes, post-laser IOP values of the treated eye of each patient were also analyzed adjusting for IOP changes (between visits variation) of the untreated fellow eye (adjusted analysis). Pre and post-laser IOP values were compared using paired *t*-test. Factors associated with the magnitude of IOP reduction were investigated using multiple regression analysis.

**Results:**

A total of 45 eyes of 45 patients were enrolled. Mean IOP was reduced from 20.8 ± 5.1 to 14.9 ± 2.9 mmHg at month 3 (*p* < 0.001). Adjusted success rate (defined as IOP reduction ≥ 20%) was 64% and mean percentage of IOP reduction was 23.1 ± 14.3% at last follow-up visit. Considering unadjusted post-laser IOP values, it was found a 20% greater absolute IOP reduction (median [interquartile range] 6 mmHg [4–7] *vs* 5 mmHg [3–7]; *p* = 0.04), with a success rate of 76%. Although baseline IOP was significantly associated with both adjusted and unadjusted post-laser IOP reduction, a stronger association was found when unadjusted IOP values were considered (*p* < 0.001 and *R* ^2^ = 0.35; *p* < 0.001 and *R* ^2^ = 0.67, respectively). Age, mean deviation (MD) index, central corneal thickness and type of glaucoma were not significant predictors (*p* ≥ 0.150).

**Conclusions:**

In this group of patients with early OAG or ocular hypertension, our short-term results confirmed SLT as a safe and effective alternative for IOP reduction. Although better outcomes were found in eyes with higher preoperative IOP, this effect was mitigated when results were adjusted to the fellow untreated eye (to the influence of between visits-IOP fluctuations).

## Background

Glaucoma is one of the most common optic neuropathies, characterized by progressive retinal ganglion cells loss, changes in the appearance of the optic disc and visual field damage [1]. Until nowadays, elevated intraocular pressure (IOP) remains the major known risk factor for glaucoma development and progression [2, 3]. Several large randomized clinical trials underscored that the only proven method to treat glaucoma is the reduction of the IOP toward a target level that will avoid functional impairment by slowing the rate of disease progression [2, 4–6]. Usually, the initial target aims for a 20 to 50% reduction on pressure, depending on baseline IOP values, disease stage and patient’s life expectancy [7]. Depending on the progression of the disease, the target pressure may need to be readjusted during follow-up [7]. Intraocular pressure may be lowered by using topical medications, incisional surgery, or laser procedures [7]. When it comes to laser surgery, the two main options available are argon laser trabeculoplasty (ALT) and selective laser trabeculoplasty (SLT) [8].

Latina and coworkers have introduced SLT in 1995, providing us with a safe and effective non-invasive treatment modality for patients with open-angle glaucoma (OAG) and ocular hypertension (OH) [9, 10]. SLT lowers IOP by inducing biological changes in the trabecular meshwork resulting in increased aqueous outflow [9, 10]. It is performed with a Q-switched Nd: yttrium-aluminum-garnet (YAG) laser (λ  =  532 nm), that delivers short burst of low-fluence laser energy to selected melanin-containing cells in the trabecular meshwork, causing intracellular targeting of the pigmented trabecular meshwork cells without damage to adjacent no pigmented cells or structures [9].

Several studies with follow-up ranging from 4 weeks to 72 months demonstrated the efficacy of SLT as an IOP lowering modality, with an average success rate of 70% [10–13]. Some recent data have suggested SLT as initial therapy, especially for eyes with early disease or high-risk OH [14]. Although substantial IOP reductions can be achieved in the majority of patients, the final SLT effect may vary significantly between patients. As approximately 30% of the patients do not respond to SLT therapy, it would be important to identify predictors of success [10–13].

In few previous reports that investigated factors associated with the magnitude of IOP reduction following SLT, the only significant predictor that has been consistently documented is baseline IOP (it is not clear until now whether this association could in part just be an effect of regression to the mean [15–17]) [10–13]. As these studies did not focus specifically on eyes with early glaucoma and most importantly did not adjust post-laser IOP results to the influence of between visits-IOP fluctuations, [10–13] we sought to determine success predictors and to study the role of the fellow untreated eye as a covariable for adjustment of IOP outcomes following SLT in early OAG or ocular hypertension patients.

## Methods

This case series study adhered to the tenets of the Declaration of Helsinki and was approved by the Institutional Review Board of the Federal University of São Paulo. Additionally, written informed consent was obtained from all participants.

### Study participants

We prospectively enrolled patients with uncontrolled early OAG or OH (inadequate IOP control requiring additional treatment). All participants underwent a complete ophthalmological examination including review of medical history, best-corrected visual acuity, IOP measurement with Goldmann applanation tonometry (Haag-Streit, Koeniz, Switzerland), slit-lamp biomicroscopy, gonioscopy, refraction and dilated fundus examination. Key Exclusion criteria were previous glaucoma surgery, history of ocular trauma or inflammation, and visual field mean deviation (MD) index worse than -6 decibels (dB).

All patients had early OAG or OH with uncontrolled IOP (individualized for each patient, based on the level of glaucomatous damage and/or based on disease progression [visual field progression confirmed by at least three visual field test or structural damage confirmed by stereophotograph]); age >18 years and no previous laser or incisional glaucoma surgery.

The definition of OAG was based on the presence of repeatable (≥3 consecutive) abnormal standard automatic perimetry (SAP) test results on the 24-2 program of the visual field (Humphrey Field Analyzer; Carl Zeiss Meditec, Inc) or if progressive glaucomatous optic disc changes were noted on masked examination of stereo photographs, regardless of the results of SAP. We defined abnormal SAP results as those with a pattern standard deviation index outside the 95% confidence limits or glaucoma hemifield test results outside the reference range. Early glaucoma was defined as characteristic OAG and reproducible visual field loss, with visual field mean deviation index better than -6 dB [18]. OH was defined as of IOP higher than 21 mmHg, with healthy-appearing optic discs and without repeatable abnormal SAP results. OH had at least three IOP measurements in each eye at pre-laser time points.

### Selective Laser Trabeculoplasty (SLT)

All participants underwent SLT (one single laser session) performed by the same surgeon in a standardized fashion. The same preoperative medical regimen was maintained during follow-up for all patients. Post-treatment assessments were scheduled at week 1 and months 1, 2, and 3. The IOP was measured with a Goldmann Tonometer.

All patients underwent to one session of SLT using a frequency-doubled Q-switched Nd:YAG laser (Laserex Tango™ Nd:YAG, Ellex Medical, Australia) emitting at 532 nm with pulse duration of 3 nanoseconds and a spot size of 400 μm coupled to a slit- lamp delivery system. A Goldmann 3-mirror goniolens was placed on the eye with 1% methylcellulose. The aiming beam was focused onto the pigmented trabecular meshwork. The 400 μm spot size was large enough to irradiate the entire anteroposterior height of the trabecular meshwork. In all eyes, approximately 100 adjacent but nonoverlapping laser spots were placed over 360° of the trabecular meshwork. Initial energy level was set to 0.80 mJ and changed according to the level of trabecular meshwork pigmentation. The end point of each laser application was minibubble formation. Brimonidine 0.2% was instilled before and after the procedure, and 0.1% dexamethasone acetate was administrated 4 times a day for 5 days in all patients.

Data collected included age, gender, race, type of OAG, visual field status, preoperative (average of 3 separate measurements) and postoperative IOP, number of antiglaucomatous medications, gonioscopy appearance, pachymetry, surgical complications, and any subsequent related event.

### Statistical analysis

Descriptive statistics included mean and standard deviation values for normally distributed variables, while those not normally distributed were presented with median and interquartile range. Skewness/Kurtosis tests and histograms were used to check Normality. Paired *t*-test was used for comparison of IOP values between each time point (baseline and post-laser treatment). For non-normally distributed variables we used a non-parametric test (Wilcoxon rank-sum test).

In order to account for possible influence of IOP fluctuation on laser outcomes, post-laser IOP values of the treated eye of each patient were also analyzed adjusting for IOP changes (between visits variation) of the untreated fellow eye (adjusted analysis). Factors associated with the magnitude of IOP reduction were investigated using multiple regression analysis.

All statistical analyses were performed with commercially available software (MedCalc software; MedCalc, Inc., Mariakerke, Belgium). The α level (type I error) was set at 0.05.

## Results

A total of 45 eyes of 45 patients were enrolled. Among these patients, 32 (72%) had primary OAG, 4 (9%) had pigmentary glaucoma, 6 (13%) had OH, and 3 (6%) had exfoliative glaucoma. Mean age and average SAP MD for this sample were 57.6 ± 11.7 years and -2.3 ± 1.8 dB, respectively. Demographic and clinical data of these patients are presented in details in Table 1. According to the Spaeth grading of pigment on the trabecular meshwork, [19] 70% patients had moderate pigmentation (PTM ++, from PTM + to PTM +++) and all patients had open angle (visible until at least the scleral spur in all quadrants during the gonioscopy). Table 2 shows details about mean IOP in each post-treatment assessment.

**Table 1:** Demographic and clinical variables of study patients (*n* = 45)

Characteristic	Value
Age (±SD), years	57.6 ± 11.7
Gender, %	M (47%), F (53%)
Race, %	W (75%), A (6%), B (12%), M (7%)
Type of Glaucoma, %	POAG (72%), OH (13%), PG (9%), EG (6%)
Baseline mean IOP (±SD), mmHg	20.8 ± 5.1 (range 12 to 39)
Non-adjusted post-laser mean IOP (±SD), mmHg	14.98 ± 2.89 (range 10 to 22)
Adjusted post-laser mean IOP (±SD), mmHg	15.71 ± 4.06 (range 7 to 31)
Number of Medications (±SD)	1.4 ± 1.2 (range 0 to 4)
Average SAP MD (±SD), dB	-2.3 ± 1.8 (range -5.9 to 0.4)
Pachymetry (±SD), μm	520.8 ± 42.6 (range 444 to 624)

*Abbreviations*: *SD* standard deviation, *M* male, *F* female, *W* white, *A* Asian, *B* Black, *M* Mixed, *IOP* Intraocular pressure, *mmHg* millimeter of mercury, *POAG* primary open angle glaucoma, *OH* ocular hypertension, *PG* pigmentary glaucoma, *EG* exfoliative glaucoma, *SAP* standard automatic perimetry, *MD* mean deviation

**Table 2:** Intraocular pressure values (mmHg) at each time point

	Baseline	1 week Post-treatment	1 month Post-treatment	3 months Post-treatment
Mean IOP (± SD) mmHg	20.8 ± 5.0	18.8 ± 5.1	16.8 ± 5.4	15.0 ± 2.9

**Fig. 1:**
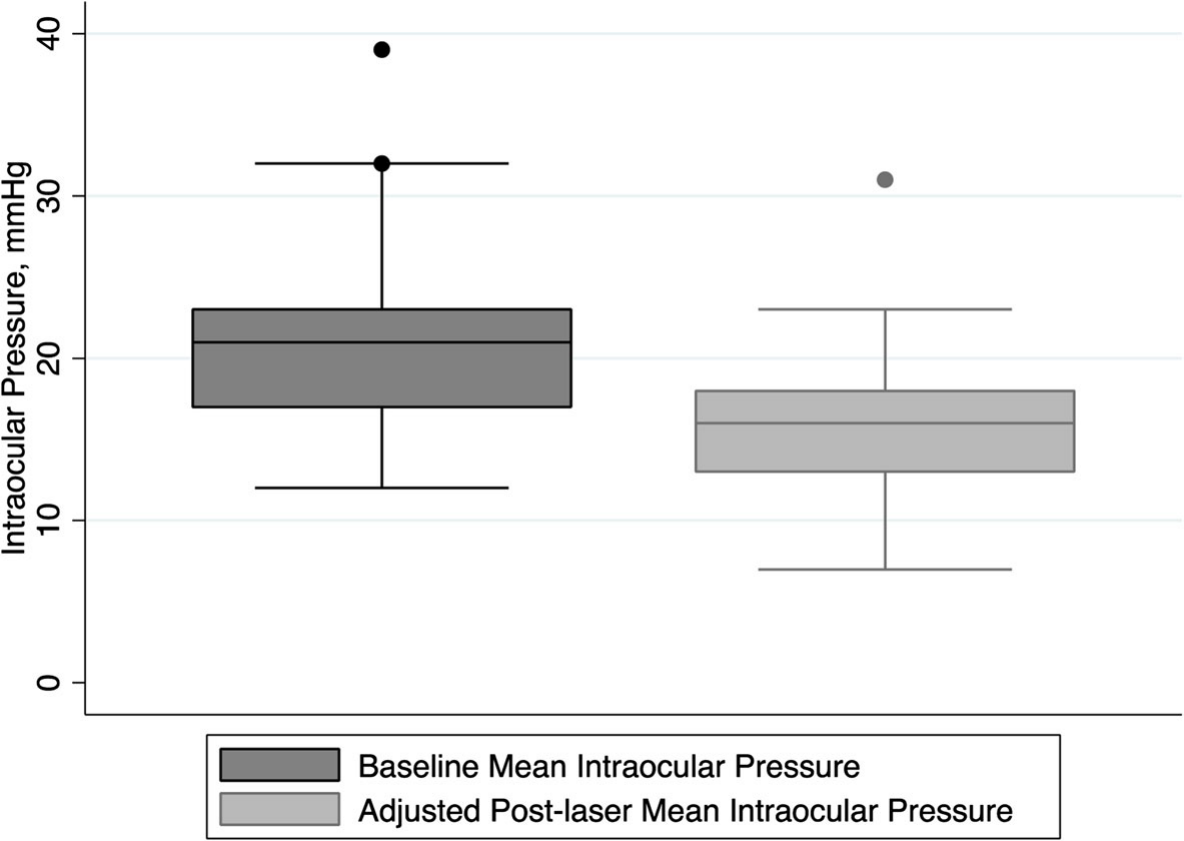
Box plots showing for adjusted success rate, the mean intraocular pressure at baseline and last follow-up visit. * Box represents median and interquartile range. Whiskers correspond to maximum and minimum 1.5 interquartile range (IQR)

**Fig. 2:**
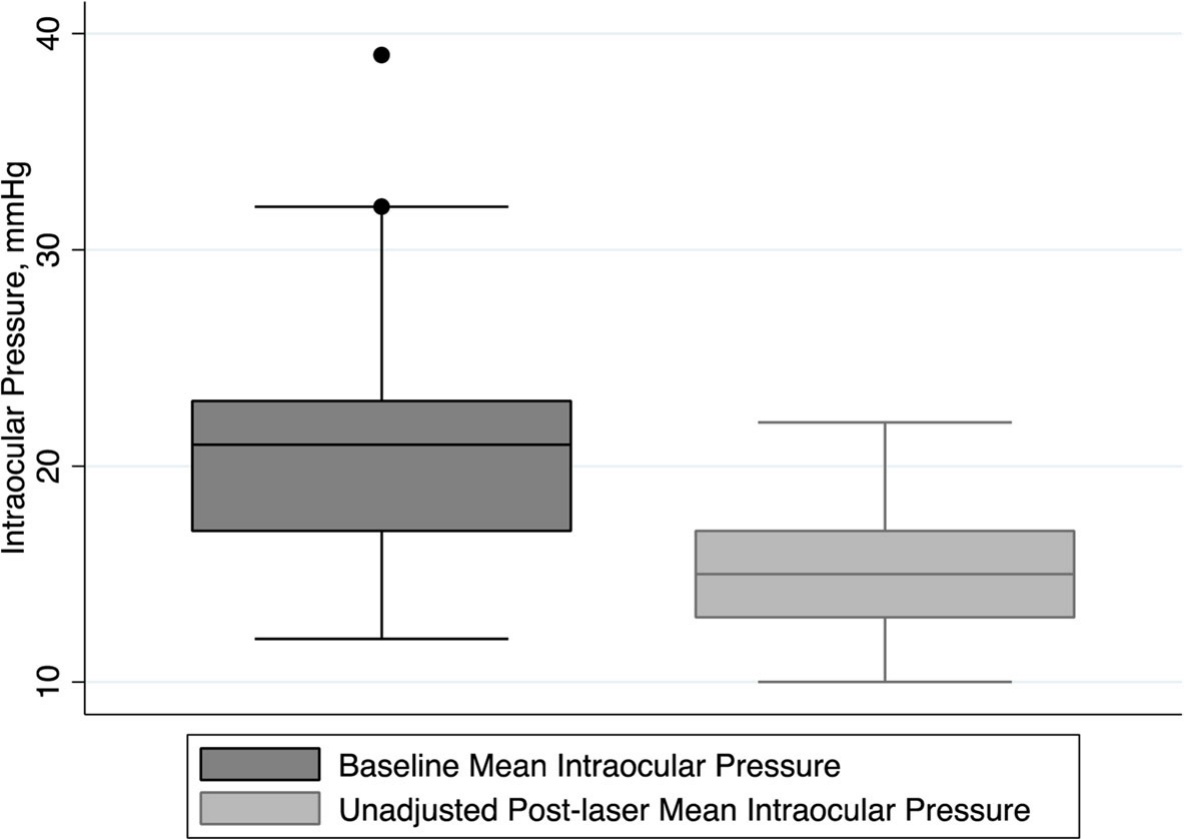
Box plots showing for unadjusted success rate, the mean intraocular pressure at baseline and last follow-up visit. * Box represents median and interquartile range. Whiskers correspond to maximum and minimum 1.5 interquartile range (IQR)

Adjusted success rate (defined as IOP reduction ≥ 20%) was 64% and mean percentage of IOP reduction was 23.1 ± 14.3% at last follow-up visit. Figure 1 shows for adjusted success rate, the mean IOP at baseline and last follow-up visit. Considering unadjusted post-laser IOP values, it was found a 20% greater absolute IOP reduction (median [interquartile range] 6 mmHg [4–7] *vs* 5 mmHg [3–7]; *p* = 0.04), with a success rate of 76%. Figure 2 shows for unadjusted success rate, the mean IOP at baseline and last follow-up. And Table 3 summarizes the laser outcomes for adjusted and non-adjusted success rate.

**Table 3:** Laser Outcomes for adjusted and non-adjusted success rates

Variables	Adjusted	Non-adjusted
Delta IOP (±SD), mmHg	5.1 ± 3.8 (range -2 to 21)	5.8 ± 3.8 (range 0 to 20)
Delta IOP (±SD), %	23.1 ± 14.3 (range -14.3 to 75)	26 ± 12.6 (range 0 to 51.3)
Success Rate, %	64%	76%

*Abbreviations*: *IOP* intraocular pressure, *SD* standard deviation, *mmHg* millimeter of mercury

In the univariable regression analysis, although baseline IOP was significantly associated with both adjusted and unadjusted post-laser IOP reduction, a stronger association was found when unadjusted IOP values were considered (*p* < 0.001 and *R* ^2^ = 0.35; *p* < 0.001 and *R* ^2^ = 0.67; respectively).

**Fig. 3:**
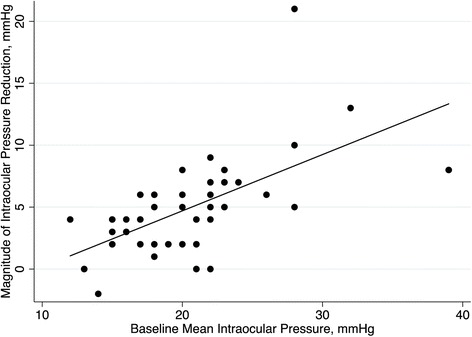
Scatter plot depicting the relationship between the magnitude of intraocular pressure reduction (adjusted values) and baseline intraocular pressure

In the multiple regression analysis, the only factor significantly associated with the magnitude of IOP reduction (adjusted values) was baseline IOP (*p* < 0.001 and *R* ^2^ = 0.46). Figure 3 illustrates the relationship between the magnitude of IOP reduction (adjusted values) and baseline IOP. Age, MD index, central corneal thickness, type of glaucoma and number of medications at the baseline were not significant for this model (*p* ≥ 0.150).

During the follow-up visits, one patient developed sustained IOP rise (≥10% IOP increase in 2 consecutive visits). There were no cases of peripheral anterior synechiae development or any other serious complication. Finally, three patients had sustained high IOP (≥21 mmHg after 3 months follow-up). Mean age and average SAP MD for these three patients were 50.5 ± 6.4 years and -2.1 ± 0.4 dB, respectively. Consequently, they were treated with eye drop medications. Incisional surgery was not required for any patient. These three patients are currently being followed with no visual field progression.

## Discussion

This study has shown that in early glaucomatous disease, SLT is safe and effective for IOP reduction. In addition, using the unadjusted analysis of post-laser IOP we tend to overestimate success rates (the influence of IOP fluctuations between the visits). To the best of our knowledge, this is the first study that provides evidence that the fellow untreated eye should be considered to allow post-laser outcomes adjustment.

Different studies have tried to determine the predictors of success for adjuvant SLT in OAG in different populations [20–25]. The different factors that have been consistently described to predict SLT success throughout the literature include: no prior antiglaucomatous medication use [22, 24, 25] and a higher baseline IOP [20, 21, 23]. This relationship between SLT success and baseline IOP has been underscored in previous publications [20, 21, 23]. Kano et al. studied 67 eyes of 67 uncontrolled OAG patients that underwent SLT and they showed that the measure of preoperative IOP was the significant determinant for success [21]. Alternatively, Damji et al. conducted a clinical trial with 36 eyes comparing SLT *vs* argon laser trabeculoplasty (ALT) and they found out that the only predictor of final IOP at 6 months was the baseline IOP [20]. In addition, in a retrospective study, Rhodes et al. observed that patients with higher preoperative IOPs had a greater reduction in IOP in both eyes [26]. In a prospective interventional study, Koucheki and Hashemi also found significant correlation between the preoperative IOP level and the IOP reduction after SLT [27]. In our study, baseline IOP was the only factor significantly associated with SLT success. Even though we have focused on eyes with early glaucoma (SAP MD better than -6 dB) or OH, our results are in agreement with those previously reported studies with glaucomatous patients with different disease stages, as they also found a positive association between baseline IOP and magnitude of IOP reduction [20, 26–28].

When we analyzed different aspects such as age, SAP MD index, central corneal thickness and type of glaucoma, no significant association was found in this present study. These findings are also in agreement with previous SLT publications that showed no correlation for sex, age, previous ocular surgery, lens status, classes of antiglaucomatous medications, angle pigmentation or type of OAG [26–29]. It is true that some studies found correlation between age and SLT success rates. For example, Ahmed et al. found that age older than 60 years was associated with greater SLT success rates and Lee et al. also described that older age was found to be a significant predictor for success (Odds Ratio: 1.11; *p* = 0.0003) [30, 31]. The reason for this disagreement may be due to different characteristics of these samples. Our patients had a mean age of 57.6 years old that is higher than the other two studies.

Regarding the association between SLT outcomes and type of glaucoma, we have not found significant association in this present study. It is true that Chen et al. showed that pigmentation at the trabecular meshwork is related to the pressure-lowering effect of SLT 7 months after the SLT treatment [32]. However, according to Hodge et al., the pigmentation of trabecular meshwork and type of glaucoma did not predict better outcome [33]. The methods used by Chen et al. included 32 patients in two different groups who received SLT with 25 laser spots on 90° of trabecular meshwork, the other 32 patients who received SLT with 50 laser spots on 180° of trabecular meshwork and were followed for 7 months post-treatment [32]. Alternatively, Hodge et al. included 89 randomized patients who were followed by 12 months post-treatment [33]. Therefore, it is possible that the different methodology applicable in different studies can lead to these different results. Future studies should be necessary to evaluate the real impact of type of glaucoma and trabecular meshwork pigmentation in SLT outcomes.

In our sample, we described one case (2%) of sustained IOP rise (≥10% IOP increase in 2 consecutive visits) that resolved without additional treatment. There were no cases of peripheral anterior synechiae development or any other serious complication. This incidence is also in agreement with reported literature. Most reported adverse effects for SLT such as discomfort, pain or photophobia are mild and resolve within few days without treatment [34, 35]. The transient IOP rise of ≥5 mmHg occurs in 0 to 28% of eyes studied, [13, 36, 37] and ≥10 mmHg in up to 5.5% of eyes [13, 23]. It usually resolves quickly with or without topical antiglaucomatous treatment, usually within 24 h. Other side effects reported in the literature are: peripheral anterior synechiae, hyphema, bilateral anterior uveitis and choroidal effusion [36, 38, 39]. There were no cases of these complications in this present study during 3-months follow-up. We also reported three patients (7%) with sustained high IOP (≥21 mmHg after 3 months follow-up). This result is not surprising, as previous studies have shown that SLT is not effective in 20–30% of the cases [13].

Moreover, the key finding of the present study was the difference between adjusted and non-adjusted post-laser results. As without accounting for the influence of IOP variation we found an overestimated lowering effect, therefore the main clinical implications of our findings is that it seems reasonable to treat one eye at a time and use the IOP values of the untreated fellow eye as controls. Probably, the association between baseline IOP and post-laser IOP results found in different previous studies is partially related to the effect of regression to the mean [15–17]. It is true that previous studies have reported a modest contralateral effect in the untreated fellow eyes of patients undergoing selective laser trabeculoplasty. Given that mechanism of effect of SLT on IOP reduction is considered to be a biological process it is possible that there is contralateral reduction in response to the SLT laser. However, this fact cannot exclude the regression to mean.

It is important to discuss some specific limitations of the present study. First, it is limited by its small sample size; however even with a small sample we found a significant difference between unadjusted analysis of post-SLT IOP and the non-adjusted one, and in addition this is the first study focused on early glaucoma patients. However, future studies should be necessary to confirm this hypothesis. Second, the 3-month IOP was used for the calculation of IOP response. A longer period following SLT would have been more ideal to estimate the real success rate. Third, topical antiglaucomatous medications were not washed out prior to SLT procedure. However, we considered unethical and inappropriate to stop antiglaucomatous medication in these patients. Additionally, as reported in the literature, the use of eye drops medication did not seem to influence SLT success significantly [28]. Lastly, we have not analyzed different aspects that could influence the SLT final results such as greater degree of spherical equivalent or more refractive error. Lee et al. recruited 51 eyes of 31 patients from Chinese population and they found that greater degree of spherical equivalent or more refractive error was a predictor of SLT success (Odds Ratio: 1.19; *p* = 0.02) [31]. Although this is an interesting point, this is not the main purpose of this study; therefore, future studies can be carried out from Brazilian population to elucidate this question.

## Conclusions

In conclusion, our results suggest that SLT is safe and effective for IOP reduction in OHT and early OAG. Higher IOP reduction was found in those with higher pre-laser IOP. We found that success was overestimated if the post-laser IOP was not adjusted for the inter visit variation in the other eye. We, therefore suggest that, whenever possible, laser should be performed in one eye at a time thus allowing for post-laser outcomes adjustment. However, analysis of visual field progression and structural examination should be always taking into account for a better understanding of the treatment impact.

## Abbreviations

ALT: Argon laser trabeculoplasty
IOP: Intraocular pressure
MD: Mean deviation
OAG: Open-angle glaucoma
OH: Ocular hypertension
SAP: Standard automatic perimetry
SLT: Selective laser trabeculoplasty
YAG: Yttrium-aluminum-garnet
